# Looking for a Person-Centered Medicine: Non Conventional Medicine in the Conventional European and Italian Setting

**DOI:** 10.1093/ecam/nep048

**Published:** 2010-12-27

**Authors:** Paolo Roberti di Sarsina, Ilaria Iseppato

**Affiliations:** ^1^Italian High Council of Health, Ministry of Health, Rome, Italy; ^2^Department of Sociology, University of Bologna, Bologna, Italy

## Abstract

In Italy, the use of non conventional medicines (NCMs) is spreading among people as in the rest of Europe. Sales of alternative remedies are growing, and likewise the number of medical doctors (MDs) who practise NCM/complementary and alternative medicine (CAM). However, in Italy as in other countries of the European Union, at the present time the juridical/legal status of NCM/CAM is not well established, mainly due to the lack of any national law regulating NCM/CAM professional training, practice and public supply and the absence of government-promoted scientific research in this field. This is an obstacle to safeguarding the patient's interests and freedom of choice, especially now that dissatisfaction with biomedicine is inclining more and more people to look for a holistic and patient-centered form of medicine.

## 1. Introduction

In western societies, people generally confuse the terms ‘medicine', ‘biomedicine', ‘evidence-based medicine' and ‘allopathic', using them as synonyms. Though the neologism ‘biomedicine' appeared as early as 1923 in *Dorland*'*s Medical Dictionary* [1]—defined as ‘clinical medicine based on the principles of physiology and biochemistry'—neither this nor other specific hybrid meanings were much heard of before the 1960s when the US National Institutes of Health (NIH) introduced the term to justify its diversion of funds into molecular biology. ‘Medicine' proper is actually something more comprehensive, a holistic concept that includes ‘biomedicine', but also all other philosophical/anthropological approaches to managing health, illness and disease. Amid the prevailing confusion, western biomedical culture tends in its turn to define ‘alternative medicine' in a negative way, as something outside the mainstream, unsupported by scientific explanation or academy legitimization [2].

## 2. The National Center for Complementary and Alternative Medicine

The National Center for Complementary and Alternative Medicine (NCCAM) of the US NIH defines CAM as a broad domain of healing resource that encompasses all health systems, practices and beliefs, other than those intrinsic to the politically dominant health system in a particular society at a given historical period. The main limitation of this definition is mixing alternative medicine and complementary practices in one single category. CAM has been defined as therapeutic intervention that has neither been widely established for use in conventional healthcare practice nor incorporated into the standard medical curriculum. The NCCAM characterizes CAM therapies into five categories: *biologically based therapies, manipulative- and body-based therapies, energy medicine, mind–body medicine and whole medical systems*. In contrast, we have the definition of traditional medicine issued by the World Health Organization (WHO) which states that traditional medicine is the sum total of indigenous knowledge used in the maintenance of health; without distinction the concept extends to *CAM, NCM, holistic medicine and natural medicine*. Such terms are used interchangeably with traditional medicine in some countries and refer to a broad set of health care practices that are not a part of that country's own tradition and are not integrated into the dominant health care system.

## 3. Other Definitions

Paradoxically, then, in western countries indigenous biomedicine and alternative medicine may come to coincide. We thus have a preference for NCM: this term is scientifically neutral and alludes to the conventional status appropriated by biomedical orthodoxy [3]. That NCM may be the first choice of people looking for well-being and health, and is not necessarily posited against the dominant paradigm [4]. The term has also been adopted by the European Parliament in its Resolution n. 75 issued on May 29, 1997 that deals with ‘The Status of NCM'.

Now, *∼*80% of the world's population uses traditional, complementary and alternative, NCM; 50% of all drugs approved were from 1981 to 2002 and 62% of anti-cancer drugs are derived from natural products. According to the WHO, more than half of the European citizens have used NCM practices and people are increasing their expenditure year by year on NCM/CAM medicines, therapies and practices. Even the number of NCM/CAM practitioners is increasing in all countries of the European Union (EU). Many MDs and medical students share their patients' uncertainty about conventional medicine and over the last 15 years have moved from a position of silent interest to one of open inquiry and growing use [5–11], while an increasing number of organized professional groups are instituting appropriate education and training.

The right of choice that today's individuals have concerning their own state of health and illness has often been stated and has taken concrete shape with increasingly large sections of the population resorting to treatments and therapeutic practices known under the umbrella: ‘*NCMs*' or ‘*CAM*' (NCM/CAM), i.e. not yet integrated into the dominant health care system.

## 4. The WHO

The WHO has always promoted traditional, complementary and alternative, NCMs. Again, the seventh Framework Programme of the European Community for research, technological development and demonstration activities (2008–13) identifies successful interventions in complementary and alternative medicine for safeguarding and improving the health of European citizens and, indirectly, their right of choice [12]. Here is the policy statement on this point runs:

*The Programme should recognize the importance of a holistic approach to public health and take into account, where appropriate and where there is scientific or clinical evidence about its efficacy, complementary and alternative medicine in its actions (European Parliament, 23.10.2007).*

According to the Italian Institute for Statistics [13], in 2005 *∼*8 million persons in Italy had used NCMs during the previous 3 years: the typical Italian user of NCM/CAM is an adult between 35 and 44 years of age with post-high school qualifications; there are a greater percentage of women; and the most used NCMs and therapies are: homeopathy, acupuncture, herbal remedies, anthroposophic medicine and chiropractic. Most users admit to having gained great or healing without side effects.

There are many explanations for the progressive diffusion of NCM/CAM therapies, which have much to offer, particularly in the absence of effective conventional approaches. Biomedicine once served to make patients better, alleviating symptoms and healing disease; now, some think it has degenerated into a risk-reducing, patient-stratifying, life-years-adding bioscience disregarding individual needs [14]. Currently, it seems that NCM/CAM can often be used as a first option in certain problems, keeping more costly, invasive and potentially toxic treatment as a second option. It may help to prevent often long-term dependency on biomedical medication and to reduce the enormous burden of mortality and morbidity caused by the iatrogenic effects of allopathic prescription drugs, peoples' ever increasing resistance to antibiotics and the inability of biomedical drugs and therapies to cope with chronic and psychosomatic diseases.

Current citizen attitudes to health include a preference for natural products instead of chemical drugs, a holistic sustainable philosophy and related behavior in terms of health management, a belief in individual responsibility for achieving wellness, more attention to relationship, dialogue and emotive needs, less unquestioning acceptance of medical authority and anti-technology feelings. Many of these arguments have been explored in the recent literature, and Roberti di Sarsina [15] emphasizes a more person-centered, humanized, personalized approach to medicine based on respect for people's whole bio-psycho-spiritual unity and equilibrium, including their relation to the environment and the way they perceive or ‘narrate' their own complex individual existence, in sickness and in health. Other works, including several geared to the Italian situation, add to the picture of current trends and perspectives [16–19].

## 5. Diffusion of NCM/CAM among Patients and Practitioners

In Europe, in spite of the increasing stringency of rules introduced by pharmaceutical agencies and European legislation, since 2003 the economic importance of homeopathic and anthroposophic medicinal products has increased by 20% in response to the demands of millions of European citizens [20]. In Italy, today there are almost 3000 MDs using acupuncture, more than 8000 homeopaths, about 20 000 MDs with homeopathic training and approximately 160 MDs who have completed the 3-year residential course on anthroposophic medicine. All these numbers are increasing, in line with European trends. NCM/CAM practitioners are joining forces in organizations designed to enhance credibility and to establish standards of practice. Almost one chemist's shop in two offers homeopathic remedies. In Italy there are 30 homeopathic laboratories, large or small, creating more and more job opportunities. According to ECHAMP (the European Coalition on Homeopathic and Anthroposophic Medicinal Products), the European turnover of homeopathic products was 810 million euros in 2007. Three out of the four Europeans know about homeopathy and of these 29% use it for their own health care. This represents 100 million Europeans.

The geographical distribution of homeopathic remedies in Italy is the following: 50% in the north, 35% in the center, 15% in the south and islands. In 1980, 2000 chemist's shops out of the 14 365, that is 14%, had a section for homeopathic remedies. By 2000, 7000 chemist's shops out of the 16 000, that is 43%, had a section for homeopathic remedies. In 2005, Italy was the third European country after France and Germany for sales of HAMP (Homeopathic and Anthroposophic Medicinal Products). About 49% of French, 46% of Germans and 35% of British use NCMs.

According to an ISTAT (Italian National Institute for Statistics) survey 2007 (relating to the year 2005) [13], 13.6% of Italians use of NCM: 4.7 million women (15.8% of Italian women), *∼*3.2 million men (11.2%), *∼*10% of the children. Most users belong to the higher social classes and live in north Italy. Among the various NCM/CAM, homeopathy proved the most common. A marked connection persisting in time is consolidated between users of such therapy and higher education. It also means that 5 years on from the previous ISTAT poll (1999) no less than 8 million Italians confirmed the worth and utility of such treatment.

The economic burden on families is another factor to be considered. As its purchasing power has been whittled away recently, the family budget is increasingly onerous to cover full costs of non-conventional health (doctor, medicines and supplementary therapy). It is workers whose categories refund such treatment (e.g. journalists, managers, etc.) that heads the list of users statistically. More and more frequently one hears cases of families where the parents reserve alternative treatment for their children and cannot afford it for themselves.

There are over 20 000 Italian practitioners prescribing homeopathic and anthroposophic medicines. Many are doctors and veterinarians who have done years of post-graduate training to acquire specific skills in homeopathy, anthroposophic medicine and homotoxicology, which are the three sectors that use homeopathic medication. Homeopathic and anthroposophic medicines are found exclusively at most Italian chemist's shops. Homeopathic medicine exists for >200 years and in Italy offers a range of more than 5000 medicines. Italy has about 30 homeophatic companies and these together employ more than 1200 persons.

Italy is the third European market after France and Germany with HAMP sales [1 and 2] and the seventh European country by HAMP consumption per head. At least 10% of Italians use homeopathic remedies (i.e. medical remedies produced with respect to relevant pharmacopeias through dilution and dynamization processes, regardless of clinical technique applied to choosing and prescribing the remedy in homeopathy, homotoxicology and anthroposophic medicine). The typical Italian user of homeopathy, homotoxicology and anthroposophic medicine tends to be an adult between 35 and 44 years of age with post-high school qualifications. The patients (70%) admit to having had benefits without side effects from the three remedies. In 2007, Italians spent some 300 million euros on homeopathic medicines. Via IVA, IRES and IRAP the State levy for 2007 was 40 million euros. Since neither homeopathic therapy nor examination by homeopaths is a burden on the state budget, homeopathy thus brings in net revenue of 40 million euros, less the saving on doctors' consultations.

## 6. Other Recent Surveys

A survey carried out in 2003 by ABACUS (institute for marketing research and opinion surveys) reveals us that 30% of Italians made regular use of NCM/CAM with good results. In the same year, DOXA (institute for statistical research and public opinion analyses) revealed that 23% of Italians, i.e. 11.5 million people, consulted homeopathic MDs. The same result was confirmed by EURISPES (Institute for political, economical and social studies) in 2006. A survey carried out by FORMAT (Institute for marketing research) on a sample group of 864 people and published in ‘Salute di Repubblica' (a weekly insert of the Italian daily *La Repubblica*) no. 284 of November 27, 2003 shows that 31.7% had used some form of NCM/CAM in the previous 3 years (23.4% in the previous year) with stable and constant benefits. Six Italians out of the 10 think that non-conventional products are effective and 45.6% find it right that the National Health System should pay for them. Nonetheless, only 27.8% of those interviewed knew that nine NCM/CAM (homeopathic medicine, phytotherapy, acupuncture, homotoxicology, traditional Chinese medicine, anthroposophic medicine, chiropractic, osteopathy and ayurvedic medicine) are recognized by the National Association of Medical Doctors and Dentists.

In 2003, a poll was carried out by the Tuscan Regional Health Agency to learn the opinions of general practitioners and pediatricians about NCM/CAM. Two of almost 3500 physicians (2228) interviewed (82% answers registered) had the following results: 15.2% use NCM/CAM; 57.8% advise their patients to use NCM/CAM; 11% have been specifically trained for NCM/CAM; 29.2% wish to be trained; 65.7% approve that NCM/CAM should be taught at the university; 23.7% have used NCM/CAM to treat their own health problems. Sixty-two per cent of the patients inform their general practitioner that they use NCM/CAM.

A study carried out on a sample of 52 332 families, to a total number of 140 011 people, and published in 2004 by Menniti-Ippolito and colleagues [21] in the Annals of the Italian National Institute for Health, reports that at least 15.6% of Italians used NCM/CAM over a 3-year span (homeopathy 8.2%, manual treatments 7.0%, phytotherapy 4.8%, acupuncture 2.9% and other NCM/CAM 1.3%). Another poll was commissioned by the monthly Italian magazine ‘Natural Style' from S&G Kaleidos Group of Milan and published in the December 2004 issue dealing with the diffusion of NCMs in Italy. The survey, performed on a sample of 500 women and men between 18 and 54 years of age, reveals that 40.8% consider NCM/CAM better than official medicine. The main reasons for choosing soft therapies are: lack of side effects (31.8%), possibility to cure oneself by restoring physical and psychic balance (27.6%), to live in a healthier way (16.9%) and to get back to nature (12.5%).

Yet, another recent survey was presented during the 2005 Erbexpo Edition held at Carrara in Tuscany. It took the form of a web poll coordinated by the team of Prof. Benigno Passagrilli, the President of the Scientific Committee of Erbexpo 2005. The identikit of the Italian NCM/CAM user is described as follows: a woman, 45 years of age, working as an employee and living in the center north approaches NCM/CAM because she shares the philosophy, tries to convince the whole family to use herbs, infusions, acupuncture and other non-conventional techniques. The survey collected 1177 answers. The result is that more women (62%) than men (38%) use NCM/CAM. The age ranges from 30 to 60 years. In particular, 30% are between 30 and 50 years old and 35% between 50 and 60 years. The elderly (60- to 70-years old) people are the least interested in the new medicines (15%), whereas the youngest are more enthusiastic users (20% between 20 and 30 years of age). With regard to professions, the majority of the 1177 interviewed are employees (28%), followed by retired people (27%). Self-employed people accounted for 17%, businessmen 11%, craftsmen 10% and other professions 7%. The survey also reveals that the use of complementary remedies involves the whole family: 68% of the people state that all the members of the family use them, in 22% of the cases only the wife, in 7% of the cases only the husband and in 3% of the cases the children. Such answers hint that on the one hand there is a strong motivation to take part in this type of poll when the family has a specific culture for natural products, and on the other hand that the children are not involved when such a background is missing. The answers suggest that the results of complementary therapies are considered good (65%), satisfactory (20%) and unsatisfactory (15%). The lack of satisfaction might indicate poor preparation on the part of the MD dealing with the patient (in Italy, we still await a law to regulate the training of medical and non-medical operators), or too high expectations by patients, due to wrong information.

By what pathways do people come to complementary medicine? Mere curiosity (21%) shares the same philosophy (48%), a desire to escape from official medicine (31%). This pattern of interest might again be read as the result of a bad doctor–patient relationship, or as anxiety about the chemical world. With regard to the information given out by the mass media, 60% of those interviewed think it is enough, 25% would like to have more and 15% consider it inadequate. Finally, an open poll proposed in two languages (Italian and English) on line by Cyber med, an online journalistic medical site, from March 2006 to September 2008 collected 87.7% of votes in favor of NCM/CAM being included in third-party insurance payment and 76.2% of votes in favor of NCM/CAM coming into the national health care system.

Looking at the data, the need for more political attention to the social demand for NCM/CAM and to the problem of regulating NCM/CAM practice becomes evident, not only to protect patients' safety and their right to choose how to take care of themselves, but also to prevent the rise of new social and geographical inequalities in the access to non-conventional therapies [1]. NCM/CAM should not remain an opportunity for the educated and rich alone but must be known and made accessible to everyone who wants to experiment ways differing from biomedicine.

## 7. Legal Status of NCM/CAM and the Need for Accredited Training

### 7.1. European Parliament

According to Ton Nicolai, President of the European Committee for Homeopathy (ECH), speaking at the European Public Health Alliance (EPHA) Conference (Bratislava, April 2007) [22], in 15 EU countries large numbers of general practitioners refer patients for NCM/CAM treatment and have a significant interest in training and information on NCM/CAM. Moreover, according to the European Coalition on Homeopathic and Anthroposophic Medicinal Products (ECHAMP), *∼*1% of all medical doctors and 40% of general practitioners in the European Union have taken training courses in a particular NCM/CAM therapy. There are professorial NCM/CAM chairs at universities in Germany, Italy, Spain, Switzerland and the UK; however, postgraduate training courses are still held mostly at private teaching centers. Various professional NCM/CAM doctor associations (ECH, ICMART and IVAA) have established training standards and accredited training courses that comply with those standards. In some EU countries, MDs can obtain specific additional qualifications in NCM/CAM issued by the national medical associations (Austria, Germany, Latvia and Romania), while in other countries the national medical associations are in favor of statutory regulation of CAM for physicians (France, Greece, Italy and Spain) [23–26].

The European Parliament (1997), the Council of Europe (1999) and the WHO (2003, 2008) have all passed resolutions calling on Member States to recognize professional qualifications and to start a national policy on NCM/CAM, but today only seven out of the 27 EU Member States have issued national policies on NCM/CAM. Some countries such as Italy regulate certain specific NCM/CAM therapies; other countries have no national policy on NCM/CAM at all. In any case, the situation remains unsatisfactory because of the failure to standardize legal status and training rules among the States [27, 28].

## 8. The Italian Republic

The Italian Republic protects health as a fundamental right of the individual, safeguards the principle of scientific pluralism and ensures that the individual be free to choose treatments and that health operators professionally qualified, with special focus on the independence of MDs as regards the choice of treatment (Suprema Corte di Cassazione, the Italian Supreme Court of Justice, 4th Section, Sentence no. 301, February 8, 2001). Italy has a national health service established in 1978 to provide uniform and comprehensive care financed by general and regional taxation (97%) and patient co-payments [29]. Under the Italian Constitution, responsibility for healthcare is shared by the State and the 20 regions; the State sets healthcare standards known as Livelli Essenziali di Assistenza (LEA)s which apply as of right to all residents throughout the country, while regions have responsibility for organizing and administering public funds (hence the many regional disparities in fiscal capacity, distribution of public facilities and appropriateness of care).

In 2002, resolution 1206 of the Council of Europe, November 4, 1999, concerning the status of NCM/CAM spurred the National Federation of Medical Doctors and Dentists (FNOMCeO) to action. By the so-called Terni Resolution, entitled ‘Guidelines on Non Conventional Medicines and Practices', nine NCM/CAM were recognized: acupuncture, traditional Chinese medicine, ayurvedic medicine, homeopathy, anthroposophic medicine, homotoxicology, phytotherapy, chiropractic and osteopathy. These are deemed to fall exclusively under the professional responsibility of medical doctors and dentists; they are ‘to all effects medical practice'. In the Professional Code of Medicine, an article (Art. 15) is devoted to NCMs. It reads as follows:

*Recourse to non-conventional practices forms an inseparable part of the profession's decorum and dignity and belongs exclusively to the direct non-delegable professional responsibility of the doctor*.

*Recourse to non-conventional practices must not divert the citizen from specific, scientifically consolidated therapies and always calls for properly informed consent. Doctors are forbidden to collaborate in any way with, or promote the practice of, third parties not of doctor status in the sector of so-called non-conventional practices*.

A similar resolution was passed in 2003 by the Italian Federation of Veterinarians (FNOVI). The range of disciplines accorded proper veterinary status, the definition of responsibility by qualified professionals and the implications for other practitioners are to all effects the same. On October 20, 2003, within the 43rd National Congress of the Italian Society of Psychiatry the first Consensus Conference of NCM/CAM in Italy was promoted, organized and chaired by Paolo Roberti di Sarsina, and a related first Consensus Document on NCM/CAM in Italy was signed by the most important associations of NCM/CAM [30]. On December 5, 2003, Roberti di Sarsina was instrumental in constituting the Permanent Committee of Consensus and Coordination for NCM/CAM in Italy chosen from among signers of the Consensus Document, with himself as coordinator. All the signatories hoped that this event would stimulate fuller integration of similar initiatives in the field of so-called basic medicine and in other fields of medical specialization.

The Government and the Parliament have been invited to pass a law acknowledging and regulating the practice of NCM/CAM. No such law has yet been passed. During the last 20 years, some 20 draft bills about NCM/CAM have been tabled without results. It can be said that Italian institutions have not yet responded to public demand; similarly, knowledge of NCM/CAM among MDs, especially general practitioners, is not as widespread as patient demand would require [31–34].

What little has been done is extremely cautious and at times even contradictory. The Supreme Court (*Cassazione*) has ruled (1982, 1999, 2003, 2005, 2007) that acupuncture is a medical practice; that whoever prescribes homeopathic products must be a doctor; that it is an abuse of the medical profession for anyone to practice NCM/CAM if they have not attained a degree in medicine. The Supreme Court clarified once and for all that public health is to be safeguarded, ruling that all NCM/CAM may only be practiced by doctors. Practice is thus subordinate to proof of attainment of the state qualifying examination, membership of the professional roll and possession in the first place of a degree in medicine.

The Constitutional Court (2005–08) has ruled that Regions may not pass laws on the recognition of professional figures or establishment of new rolls; these offices are reserved for the State. On the question of the constitutional legitimacy of regional legislation to regulate professional activity, the Constitutional Court ruled that such regional legislative power in matters pertaining to the ‘professions' must abide by the principle whereby the recognition of professional figures and respective qualifications is reserved for the State for necessary reasons of uniformity; it falls to the regions to discipline features that have a specific connection with the region itself. This principle applies not only to establishing particular specific norms, but also as a general limit beyond which regional law may not step. It thus follows that such laws as have been submitted for approval by the Law Judge are constitutionally illegitimate.

In 2006, Italy received the European Directive on Drugs 2004/27/CE that contains five specific articles on homeopathy and anthroposophic medication taking account of the peculiar production and control features relating to these two classes of drugs. That the Italian government has received the Directive means, at least, that such homeopathic and anthroposophic drugs as exist on the Italian market are legitimate until 2015. There is, however, an unaccountable failure as yet to introduce a series of norms to safeguard the citizen. For example, there is a law (which only Italy possesses) that bans printing instructions and dosages on the packet (to the serious detriment of the consumer). Another incredible fact: for many years, new homeopathic medicines have not been authorizable. Ever since 1995, the administrative procedure for registering new drugs has been in abeyance. Even publicizing homeopathic remedies is banned in any form, which clears further evidence of discrimination. After the procrastination for 20 years, Parliament is therefore urged to legislate without further delay and approve a full-scale national law on NCMs. In April 2009, the AIFA (the Italian drug agency) issued the first guidelines on the quality of homeopathic products, improving in this way the therapeutic opportunities even in terms of pharmacoeconomy [35].

Meanwhile, one obvious implication of state recognition for the practice of NCM/CAM as a medical act is that the State itself should promote professional training and provide information for students of university faculties such as medicine and surgery, veterinary studies, dentistry and all other connected faculties. There should also be post-graduate training with specialization courses or masters similar to those already existing in several Italian universities (Milan, Palermo, Rome, L'Aquila, Chieti, Brescia, Florence, Pisa, Verona and Bologna). The State should also acknowledge the training activity carried out during recent years by private associations and schools in this sector. It should be stressed that this kind of higher education, based on holistic paradigms and philosophies, not only regards methods and practices, but also addresses the human relationship between doctor and patient and seeks to improve the personalization of care.

According to the Parliament, the most important thing is to guarantee patients the possibility to choose non-conventional therapies, by providing accurate and complete information, thus allowing free choice of individual health paths. On March 19, 2008, the Italian Ministry of Health signed a ministerial decree including NCMs among the medical practices covered by integrative insurance funds. These public funds are to cover NCM/CAM services that are not included among the LEAs (basic welfare levels) if provided by public or private accredited structures acting officially on behalf of the National Health Service.

## 9. Regional Initiatives

Important initiatives have recently been taken by many regions after the reform of Clause V of the Italian Constitution, to the effect that the Regions should bear joint legislative jurisdiction with regard to the ‘professions'. Since the lack of a national law governing NCM/CAM makes access to these practices unequal [36], some regions such as Tuscany have included a chapter on NCM/CAM in their regional health plans: acupuncture is guaranteed as an approved regional LEA, patients are to pay a basic contribution for homeopathy, phytotherapy, acupuncture and traditional Chinese medicine services, and these are already available in 63 regional welfare centers at specially low-controlled prices.

### 9.1. Italian Regions

In Campania by resolution no. 3589 dated December 2003 and with Guidelines as to the allocation of research funds and the form of support by the ASLs (local health agencies, public enterprises that are legally offshoots of the region) and by private centers already operating in the regional territory (D.P.G.R. no. 1182 dated 15/11/2001), the Region allocated a restricted fund of 3 000 000 euros to NCM/CAM, which was later increased to 4 000 000. The fund covered NCM/CAM services offered as Basic Levels of Regional Assistance (LEAs) and training activity on NCM/CAM.

In the Emilia-Romagna Region, resolution no. 297 dated February 23, 2004, established an official health board Regional Observatory for NCM/CAM responsible for setting up and promoting experimental projects to be run by the Local Health Agencies, focusing in particular on acupuncture, homeopathy and phytotherapy, the aim being to find solutions for the integration of NCMs within biomedicine. In 2006, the Region of Emilia-Romagna sent Parliament a proposed national bill to regulate CAM. This is the first time an Italian Region has used the prerogative foreseen by art. 121 Comma 2 of the Italian Constitution.

Umbria focuses on training for MDs who practice NCM/CAM in public surgeries and has defined specific tariffs and access pathways. With a slightly different emphasis, the Lombardy Region's past ‘legislatures' have seen more than 10 experimental and observatory studies and clinical audits on NCM/CAM, promoted by both public services and private institutes in collaboration with the WHO, which have now reached the operating stage.

### 9.2. Italian Perspectives

Despite these positive signals, in Italy there is still no mature culture of NCM/CAM or any shared awareness of the steps that should be taken to regulate this world. In the absence of national regulations, the Regions do not act uniformly or promote action leading to harmonization of decisions and behavior. Until the Italian Parliament issues a national law on NCM/CAM, the regions are the main actors in the process of management, authorization and recognition of NCM/CAM in Italy.

## Figures and Tables

**Figure 1:**
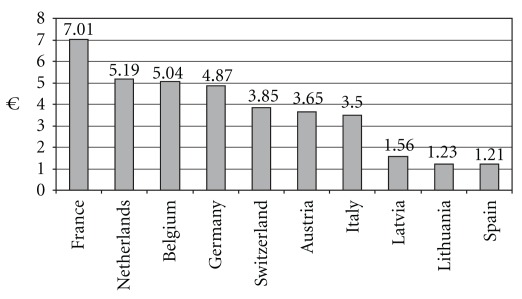
Top 10 European countries by HAMP consumption per head in € (source: ECHAMP, 20).

**Figure 2:**
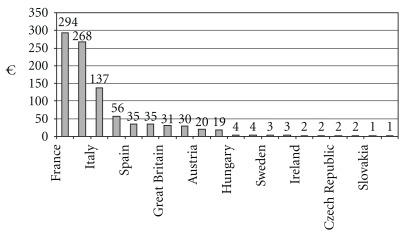
European sales of HAMP by country 2005 (source: ECHAMP, 20).

**Table 1:** Results of most recent research in Italy

ISTAT (Italian National Institute for Statistics) (1996–99): 9 million (15.5%)
ABACUS (Institute for Marketing Research and Opinion Surveys) (2003): 30% population
DOXA (Institute for Statistics Research and Public Opinion Analyses) (2003): 23% population
ISPO Research Institute (2003): 65% familiar with the term NCM/CAM and know about them
FORMAT (Institute for Marketing Research) (2003): 31.7% use NCM/CAM at least once; 23.4% use NCM/CAM regularly
CENSIS (Centre for Social Researches) (2003): *∼*50% consider NCM/CAM useful; >70% ask for National Health System reimbursement; 65% want more control by national health authorities
Menniti-Ippolito *et al.* [21]: 3 year follow-up of 52 332 families (140 011 people), use of NCM/CAM by Italian population: 15.6% (homeopathy 8.2%, manual therapies 7.0%, phytotherapy 4.8%, acupuncture 2.9% and other NCM/CAM 1.3%)
ISTAT (Italian National Institute for Statistics) (2005): 8 million (13.6%) population
EURISPES (Institute for Political, Economical and Social Studies) (Rapporto Italia 2006): 11 million use homeopathic medicine
CENSIS (Centre for Social Researches) (2008): 23.4% have used NCM/CAM during the previous year (especially homeopathy and phytotherapy)
